# A *Naganishia* in high places: functioning populations or dormant cells from the atmosphere?

**DOI:** 10.1080/21501203.2017.1344154

**Published:** 2017-07-12

**Authors:** Steven K Schmidt, Lara Vimercati, John L Darcy, Pablo Arán, Eli M.S Gendron, Adam J Solon, Dorota Porazinska, Cristina Dorador

**Affiliations:** a Department of Ecology and Evolutionary Biology, University of Colorado, Boulder, CO, USA; b Laboratorio de Complejidad Microbiana y Ecología Funcional, Instituto Antofagasta & Centre for Biotechnology and Bioengineering (CeBiB), Universidad de Antofagasta, Antofagasta, Chile; c Departamento de Biotecnología, Facultad de Ciencias del Mar y Recursos Biológicos, Universidad de Antofagasta, Antofagasta, Chile; d Molecular, Cellular, and Developmental Biology Department, University of Colorado, Boulder, CO, USA

**Keywords:** Llullaillaco, Atacama, volcanoes, astrobiology, Cryptococcus, Antarctica

## Abstract

Here, we review the current state of knowledge concerning high-elevation members of the extremophilic *Cryptococcus albidus* clade (now classified as the genus *Naganishia*). These fungi dominate eukaryotic microbial communities across the highest elevation, soil-like material (tephra) on volcanoes such as Llullaillaco, Socompa, and Saírecabur in the Atacama region of Chile, Argentina, and Bolivia. Recent studies indicate that *Naganishia* species are among the most resistant organisms to UV radiation, and a strain of *N. friedmannii* from Volcán Llullaillaco is the first organism that is known to grow during the extreme, diurnal freeze-thaw cycles that occur on a continuous basis at elevations above 6000 m.a.s.l. in the Atacama region. These and other extremophilic traits discussed in this review may serve a dual purpose of allowing *Naganishia* species to survive long-distance transport through the atmosphere and to survive the extreme conditions found at high elevations. Current evidence indicates that there are frequent dispersal events between high-elevation volcanoes of Atacama region and the Dry Valleys of Antarctica via “Rossby Wave” merging of the polar and sub-tropical jet streams. This dispersal hypothesis needs further verification, as does the hypothesis that *Naganishia* species are flexible “opportunitrophs” that can grow during rare periods of water (from melting snow) and nutrient availability (from Aeolian inputs) in one of the most extreme terrestrial habitats on Earth.

## Introduction

Recent work in the Atacama Desert Region of Chile and Argentina has shown that some of the highest elevation (>6000 m.a.s.l.) soil-like environments on Earth are dominated by basidiomycetous yeasts in the former *Cryptococcus albidus* clade most closely related to *Naganishia friedmannii* (Costello et al. 2009; Lynch et al. 2012; Solon et al. 2017). In addition, these same yeasts are fairly easy to isolate from soils at elevations from 5000 (Pulschen et al. 2015) to over 6000 m.a.s.l. (Vimercati et al. 2016) in the Atacama region. These findings are somewhat surprising given the extreme dryness of these sites and the fact that they contain some of the lowest levels of total carbon (<0.03%) of any soil environment yet studied on Earth (Schmidt et al. 2012). In addition, high elevation sites in the Atacama region have a thin atmosphere, extreme aridity, high levels of UV radiation, low pH values, and extreme diurnal freeze-thaw cycles (Schmidt 1999; Lynch et al. 2012; Cabrol et al. 2014; Pulschen et al. 2015). This unique combination of environmental stressors makes these high-elevation sites ideal for studying the dry-cold limits to life on Earth, as well as offering one of the best Earth analogues for the surface and near sub-surface of Mars (Lynch et al. 2012; Pulschen et al. 2015).

The extreme nature of high-elevation sites in the Atacama region brings into question whether *Naganishia* species can grow in this environment or if they are just dormant propagules that fall onto these remote slopes from the atmosphere. The goal of this paper is to present evidence for and against a functional role for *Naganishia* species in extreme high-elevation and high-latitude ecosystems. More specifically, it is argued that fungi in the genus *Naganishia* have adapted to be transiently functional during brief periods of water and nutrient availability in extremely dry, oligotrophic volcanic soils. Their unique adaptations include a resistance to high doses of UV radiation, adaptation to low pH values, the ability to grow at temperatures below 0°C, and most importantly their ability to grow during the extreme freeze-thaw cycles that occur on a daily, year-round basis at elevations above 6000 m.a.s.l.. This paper is a review of the current state of knowledge concerning the distribution and ecological tolerances of members of the genus *Naganishia* found at elevations from 5000 to over 6000 m.a.s.l. in the Atacama region, and compares them to other extreme high-elevation sites and to the well-studied *Naganishia* species from the Dry Valleys of Antarctica. Broader and more general discussions of the biogeography of yeasts found in cold environments are available in the literature (e.g. Vishniac 2006; Buzzini et al. 2012).

## Phylogeny of the *Naganishia* clade

Determining precise phylogenetic relationships is crucial to our understanding of the ecology of widely dispersed organisms such as *Naganishia* species. The present study benefits greatly from multiple phylogenetically relevant genes having been sequenced for the extremophilic isolates and environmental samples discussed in the present paper. The unofficial type species for extremophilic *Naganishia* species is *N. friedmannii* (Vishniac 1985a) which was originally isolated from Antarctic cryptoendolithic samples described by Friedmann (1982). Later sequencing efforts showed that *N. friedmannii* and other Antarctic (e.g. *N. antarcticus, N. consortionis*, and *N. vishniacii*) and Himalayan (*N. bhutanensis*) cryptococci grouped in a clade with *N. albidus* (Takashima and Nakase 1999). This grouping has been confirmed by numerous other studies including the recent work of Liu et al. (2015a, 2015b) who showed that *N. friedmannii, N. antarcticus, N. vishniacii* and *N. bhutanensis* are in the albidus clade of the Filobasidiales based on a seven-gene (RPB1, RPB2, TEF1, CYTB, and 3 rDNA genes) phylogeny. Also relevant to the present study, Vimercati et al. (2016) showed that the isolate from Volcán Llullaillaco (discussed below) forms a monophyletic clade with *N. friedmannii* based on long-read 18S environmental sequences (1739 bp, JX099190) from Llullaillaco (Lynch et al. 2012). Likewise, the *Naganishia* isolate from Volcán Saírecabur studied by Pulschen et al. (2015) showed 100% identity with *N. friedmannii* over a 599 bp region of the LSU rRNA gene.

## The environmental and biogeochemical setting

**Figure 1.**
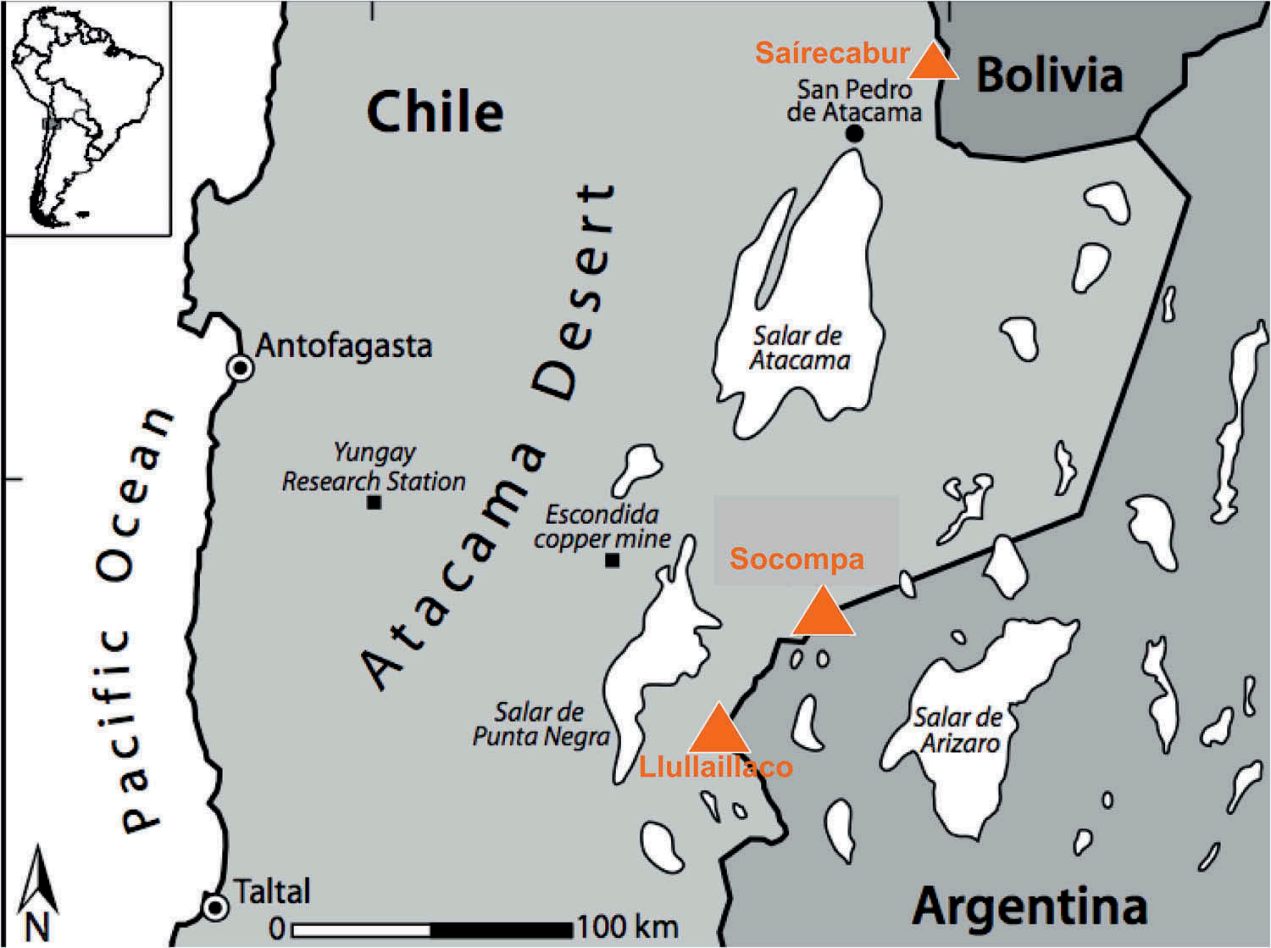
Map showing the location of the three volcanoes discussed in this paper. From south to north, microbial communities on Llullaillaco (6723 m) and Socompa (6051 m) have been studied extensively via culture-independent and culturing approaches (Costello et al. 2009; Lynch et al. 2012, Vimercati et al. 2016; Solon et al. 2017), whereas *Naganishia* and other yeasts have been cultured (Pulschen et al. 2015) from moderately high slopes of Saírecabur (5971 m). Map is redrawn from Costello et al. (2009).

**Figure 2.**
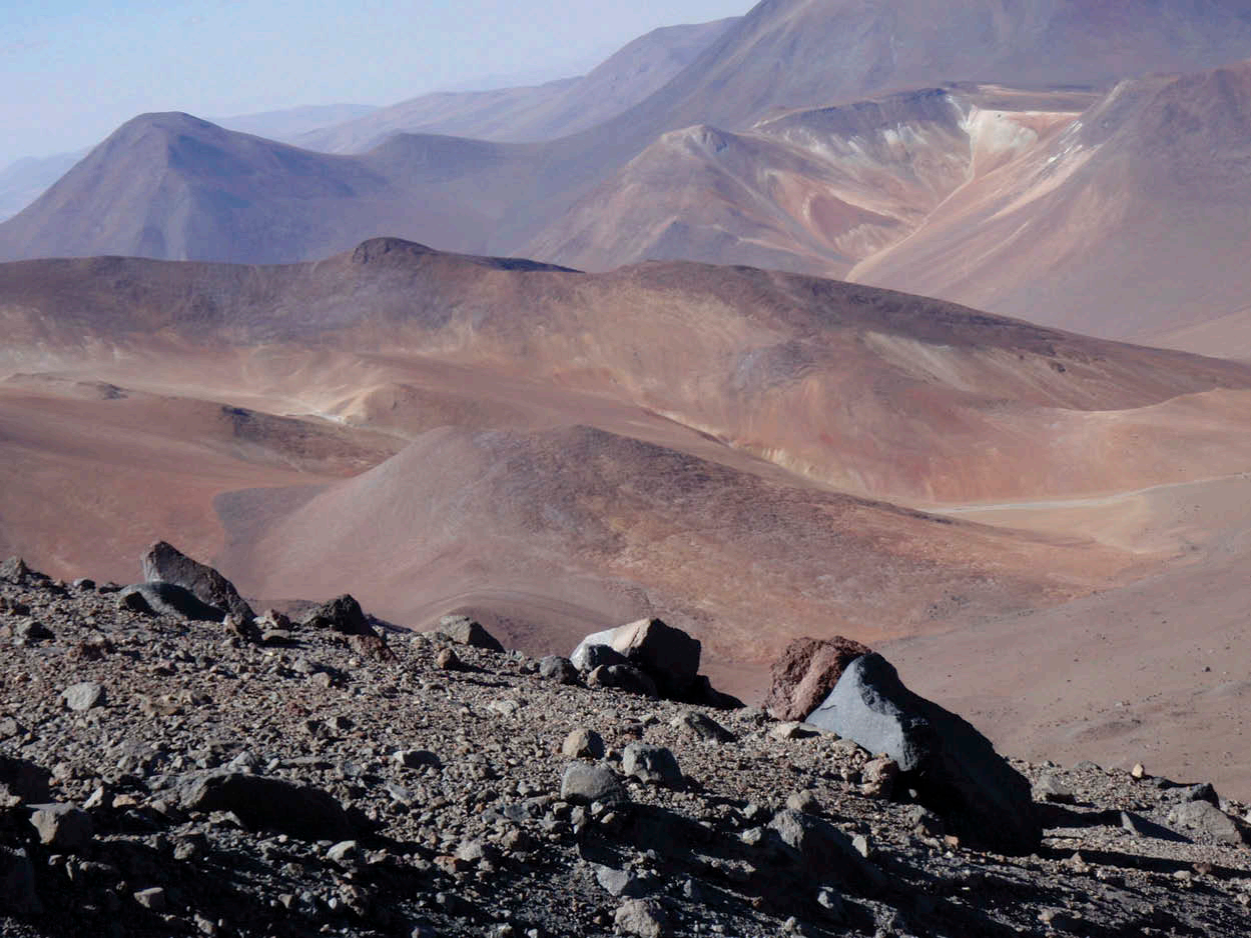
Photograph taken on Volcán Llullaillaco in February, 2009 looking down from 5800 m.a.s.l. to the landscape between Llullaillaco and Socompa. The immediate foreground shows the Mars-like tephra material (at 5800 m.a.s.l.) dominated by *N. friedmannii* OTUs.

**Figure 3.**
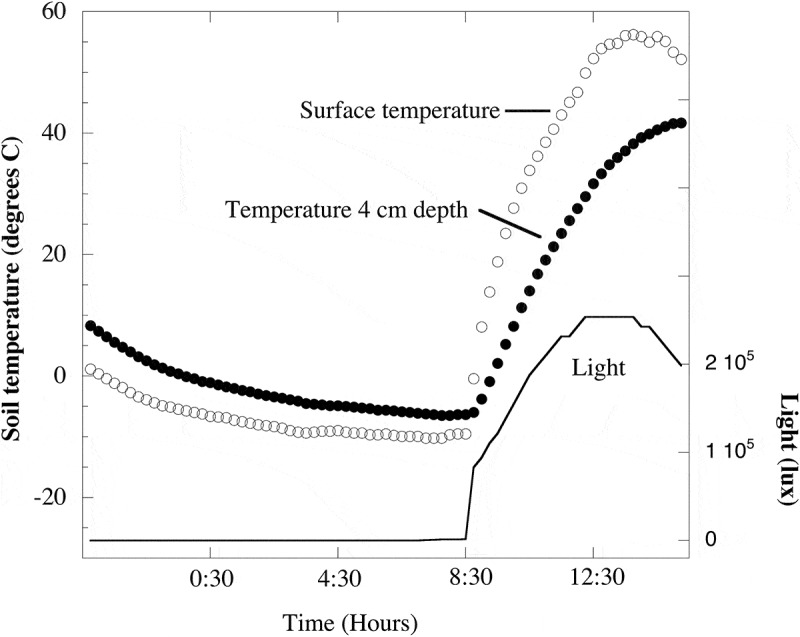
Soil temperatures and light measured at 5500 m.a.s.l. on Volcán Socompa during a summer night and day (Feb. 10 and 11, 2009). Soil surface temperatures ranged from −10.2°C at 7:30 h to a high of 56.2°C at 13:45 h. Temperatures at 4 cm depth ranged from −6.5°C at 7:45 h to 41.7°C at 15:15 h. No light penetrated to 4 cm depth and light at the surface reached the maximum measurable levels (253,512 lx) for a period of 1.75 h starting at 12:15 h. Temperature data were originally reported in Lynch et al. (2012) and light data are previously unpublished.

This paper focuses on recent work conducted in what is perhaps the most extreme terrestrial environment on Earth, that is, the highest reaches of massive stratovolcanoes in the Atacama region of Chile, Bolivia, and Argentina (Figure 1). Many studies have focused on the low-elevation areas of the Atacama Desert (e.g. Arroyo et al. 1988; Richter and Schmidt 2002; Drees et al. 2006; Demergasso et al. 2010; Neilson et al. 2012). However, very little biological work has been done in the remote, high-elevation, unvegetated zone that exists above 5100 m.a.s.l.. The highest reaches of these volcanoes receive limited snowfall, most of which sublimates back to the atmosphere resulting in some of the driest soil-like material (tephra) yet studied on Earth (Table 1, Figure 2). In addition, the thin atmosphere combined with intense solar radiation impinging on volcanic tephra creates daily freeze-thaw cycles at the soil surface (−10 to +56°C on a single summer day, Figure 3). Levels of UV radiation are very high at these sites; UVA and UVB levels at 5091 m.a.s.l. are 25–33% higher than levels measured at lower elevation sites in the Atacama (Yungay, 948 m.a.s.l.; Pulschen et al. 2015). Also of relevance to this paper is the fact that the tephra soils on these high volcanoes are among the most oligotrophic soils or sediments yet studied. Concentrations of carbon and nitrogen are at or below detection limits (Table 1), even though deep sequencing of DNA in soils from 6030 m.a.s.l. indicates that there is foreign (Aeolian) plant DNA from lower elevations being deposited there (Vimercati et al. 2016). In addition, these sites are all acidic, with pH values of 4.1–5.4 (Table 1), adding yet another dimension of stress to organisms living in these soils.

**Table 1.** Biogeochemical characteristic of soils from the three volcanoes and the Colorado site (Navajo Peak) discussed in this paper. Note the low pH values at these sites.

Mountain (el.)	elevation m.a.s.l.	pH	% C	% N	% H_2_O	ref
Llullaillaco	6034	4.2	0.017	<dl^a^	0.24	Lynch et al. 2012
	6330	4.6	0.005	<dl	0.25	Lynch et al. 2012
Saírecabur	3981	5.4	nd^b^	nd	nd	Pulschen et al. 2015
	5047	4.3	nd	nd	nd	Pulschen et al. 2015
Socompa	5235	5.2	0.03	<dl	<dl	Costello et al. 2009
	5820	5.1	0.06	<dl	3.4	Solon et al. 2017
	6030	4.8	nd	nd	0.67	Solon et al. 2017
Navajo	3878	4.5	0.85	0.09	10.7	King et al. 2010

^a^below detection limits

^b^not determined

## Global aerial dispersal of *Naganishia* species

The presence of foreign plant DNA in the plant-free areas on these volcanoes (Vimercati et al. 2016) suggests that even the microbes in these soils may just be dormant propagules from far off locations. Many microbes can be transported over global distances (Kellogg and Griffin 2006; Schlesinger et al. 2006), and biogeographic patterns of microbial community structure suggest widespread long-distance dispersal at global scales (Barberán et al. 2007, Darcy et al. 2011; Itani and Smith 2016). Mathematical models of long-range transport also show that smaller particles – like spores – are likely to easily attain global distribution (Norros et al. 2014, Wilkinson et al. 2012). But not all microbes can survive aerial transport, since the atmosphere itself is an extreme environment with stressors including high UV radiation, extreme temperatures, and high potential for desiccation (Griffin et al. 2001).

There is evidence for the survivability of members of the genus *Naganishia* in the upper trophosphere and perhaps even higher in the atmosphere. For example, *N. albida* has been isolated from filtered air samples from 2,700 m.a.s.l. in the Northwest USA (Smith et al. 2012) and from 1,464 m.a.s.l. in Western Europe (Amato et al. 2007), showing that these fungi are still viable after aerial transport. From a culture-independent perspective, *N. albida* and other *Naganishia* OTUs were detected in Israeli dust storms from 2012 and 2013 (Katra et al. 2014). Similar dust events have been tracked from Asia to North America indicating a potential route for dispersal of *N. albida* through the atmosphere (Griffin et al. 2001; Smith et al. 2012). Whether or not these organisms are active in the air column is unknown, but there is the potential for global dispersal of members of *Naganishia* species via wind and cloud formations in the troposphere. Members of the *N. albida* clade have also been found in the International Space Station where high levels of ionising radiation are a persistent stressor (Dadachova and Casadevall 2008).

Other yeast in the Tremellomycetes (including human pathogens) also show the potential for global atmospheric dispersal. For example, *Cryptococcus gatti* was traditionally considered to be restricted to tropical and subtropical climates; however, there is increasing evidence that it can be dispersed via the atmosphere even to colder areas such as the Northwest USA (Kidd et al. 2004; Byrnes et al. 2010). Other investigations into the ecological niches of these opportunistic pathogens have revealed a global distribution (Chen et al. 2014) often associated with reservoirs such as guano (Nielsen et al. 2007), Eucalyptus trees (Ellis and Pfeiffer 1990a), and *Cassia* trees (Lazera et al. 2000; Granados and Castañeda 2005). Furthermore, air sampling in Australia during the flowering season of *Eucalyptus camaldulensis* contained *C. gattii* (Ellis and Pfeiffer 1990b) as did air samples collected after tree-cutting activities in Canada (Kidd et al. 2007). Finally, Casadevall et al. (2003) identified several virulence factors of *C. neoformans* that are “dual use” with both an environmental survival function and a pathogenetic function: a capsule that provides desiccation resistance as well as production of melanin for UV shielding, heat tolerance, and cold tolerance. All three factors would enhance survival during atmospheric transport.

There is also an open question of whether the small (1–2 µm in diameter), easily dispersed sexual spores are able to survive long-distance dispersal or whether it is only whole cells that are being dispersed long distances. For most pathogenic *Cryptococcus* species, sexual spores are only known to be produced in the laboratory and have not been found in nature or in clinical samples (Velagapudi et al. 2009); however, Nielsen et al. (2007) found that *Cryptococcus neoformans* can mate on pigeon guano. To our knowledge, no extremophilic members of the genus *Naganishia* are known to have a sexual stage (Barnett et al. 2000), but this could just be because the source habitat (reservoir) for this group has not yet been discovered. There is a chance that the extremophilic members of the genus *Naganishia* are being dispersed from some as yet undiscovered source habitat where sexual reproduction can occur. It is intriguing in this regard that there is a recent report of a pathogenic strain of *N. friedmannii* (Ekhtiaria et al. 2017) – perhaps indicating that we are only just starting to understand the global reservoirs for extremophilic members of the genus *Naganishia*.

The search for a possible global reservoir for *Naganishia* species is an important goal for future studies. Our working model is that the reservoir is the high elevation volcanoes discussed in this paper, because that is where we find the highest relative abundance of members of this group (see next section) on a global scale. The relative abundance of *Naganishia* species in microbial communities of the Dry Valleys of Antarctica is less clear because most studies have been based on culture-dependent approaches focusing solely on specific species (Vishniac and Hempfling 1979; Vishniac 1985a, 1985b, 2006). Two recent culture-independent studies of microbial communities indicated that *Naganishia* are rare in many Dry Valley soils (Fell et al. 2006), but are quite common in several higher elevation sites (Dreesens et al. 2014). It is possible that the source for the *Naganishia* species found in Antarctica is the continuous dispersal of dust and particles from the Southern Andes via meandering Rossby waves in the troposphere (Madden 1979). These waves result from the confluence of the polar and subtropical jet streams which join and separate at periodic intervals (Ambrizzi et al., 1995; James 1988; Polvani and Saravanan 2000) and could drive the aerial dispersal of microbes in both directions between the Andes and Antarctica. Visualisation of Rossby waves can be found online (http://squall.sfsu.edu/crws/archive/jetstream_archive.html).

## Landscape patterns of *Naganishia* distribution

Environmental sequencing efforts using three different primer sets and two different sequencing technologies all indicate that high-elevation tephra on Socompa and Llullaillaco are dominated by phylotypes closely related to *N. friedmannii*. The first report showing this dominance came from the culture-independent study of Volcán Socompa conducted by Costello et al. (2009). They collected tephra samples (0 to 10 cm depth) in March, 2005 along a 1-kilometre transect at 5,235 m.a.s.l. and combined the samples to make a clone library using Sanger sequencing of the 18S rRNA gene (primer pair 515F and 1391R). This library was overwhelmingly dominated (61%) by *Naganishia friedmannii* and included only six other 18S OTUs (Costello et al. 2009). On a subsequent expedition in February 2009, samples (0 to 4 cm depth) were collected at six locations spanning elevations of 6030 to 6330 m.a.s.l. on the Argentinian side of Volcán Llullaillaco, and elevations of 5820 to 6031 m.a.s.l. on Volcán Socompa. The Llullaillaco samples were Sanger sequenced using primer pair 4Fa-short and 1492R and yielded high-quality, long-read sequences of which 92% fell into a single OTU (97% identity) most closely related to *N. friedmannii* (Lynch et al. 2012). The 2009 Socompa samples were sequenced using Ilumina MiSeq (primer set 1391F/EukBr) and showed overwhelming dominance of *Naganishia* in barren soils at 5820 and 6031 m.a.s.l. on Socompa (Solon et al. 2017). Finally, numerous samples were collected in 2016 across a range of elevations (5100 to 5600 m.a.s.l.) on the Chilean side of Llullaillaco and again showed an overwhelming dominance of all dry sites by *Naganishia* (Solon et al. 2017). In addition to culture-independent studies, *N. friedmannii* has been isolated into pure culture from sites at 6030 m.a.s.l. on Llullaillaco (Vimercati et al. 2016) and sites at 3981 and 5047 m.a.s.l. on Volcán Saírecabur (Pulschen et al. 2015). The ecological tolerances of these isolates are discussed in the next section (below).

The complete dominance of *N. friedmannii* at all dry sites on Llullaillaco and Socompa led us to examine environmental sequence data sets from a number of other extreme high-elevation sites throughout the world. These included 18S data sets from unvegetated sites in the high Himalayas (Schmidt et al. 2011), the high Andes of Peru (Nemergut et al. 2007), Denali National Park, Alaska (Darcy and Schmidt 2016), Mt Kilimanjaro (Vimercati et al. unpubl.), and along the continental divide in Colorado. Out of these environmental sequencing surveys significant numbers of *Naganishia* species were only found at the highest sites sampled in Colorado (Freeman et al. 2009). Importantly, these sites were also the only sampled sites (among the global sites listed above) that had soil pH values (pH 4.5) similar to those found on the three volcanoes (Table 1). All the other sites (Alaska, Peru and Nepal) had soil pH values above 7.5 (Nemergut et al. 2007; Schmidt et al. 2011; Darcy and Schmidt 2016). On-going work at the Niwot Ridge LTER site in Colorado and at sites on Volcán Llullaillaco are attempting to parse out the effects of elevation versus pH on the landscape distribution patterns of *N. friedmannii* (Gendron et al., unpublished data). It is also possible that many of the *Naganishia* species isolated from alkaline sites across the Antarctic Dry Valley landscape actually originated in acidic microsites. De Los Ríos et al. (2003) showed that endolithic cyanobacterial communities contain acidic microsites and many Dry Valley *Naganishia* species were isolated from soils downhill from endolithic sites (Vishniac 1985b) or directly from endolithic samples (Friedmann 1982; Vishniac 1985a). Obviously more work is needed to understand the factors controlling the landscape and global distribution of extremophilic *Naganishia* species.

## Ecological tolerances of *Naganishia*

The fact that the genus *Naganishia* dominates culture-independent surveys of high-elevation volcanic sites and that these yeasts are easy to isolate from such soils, does not prove that they are actually functioning *in situ*. An alternative hypothesis is that they are just dormant propagules that are being globally distributed to high elevations (like many bacteria) and are extremely adept at surviving in a dormant state (see discussion above). The sequencing and culturing approaches used so far to study these volcanoes cannot distinguish between dormant and active cells; that is, dormant cells are sequenced in environmental DNA libraries and also can germinate when exposed to rich laboratory media, even if they are not active in the environment they are isolated from. One approach to resolving this issue would be to use transcriptomic approaches, but such methods are very hard to implement in extremely remote, oligotrophic, low-biomass systems. A less direct approach is to determine whether the organisms that are cultured from these systems are able to function and grow under realistic extreme conditions in the laboratory.

**Figure 4.**
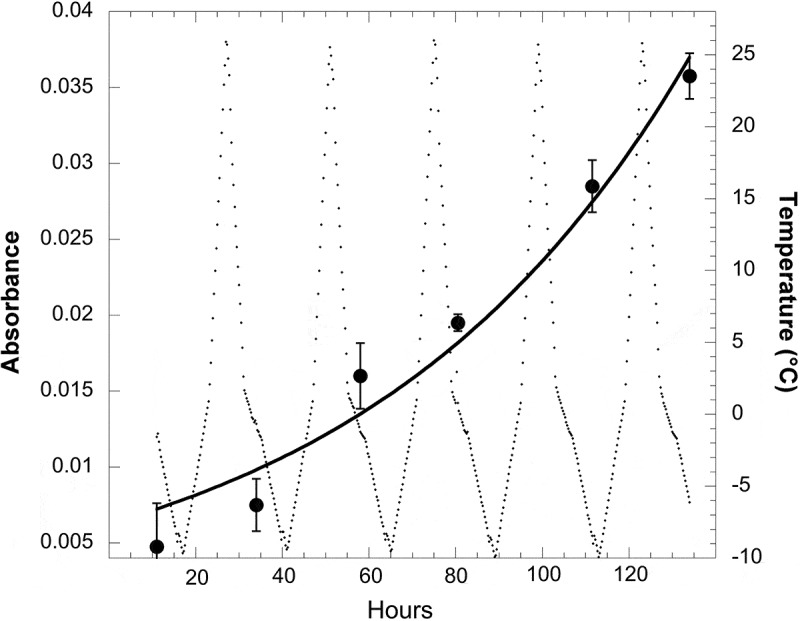
Growth of the *N. friedmannii* isolate from Volcán Llullaillaco during five consecutive freeze-thaw cycles in a specially designed freeze-thaw chamber (Vimercati et al. 2016). Growth of the cultures (4 replicates, large dots) was measured using absorbance at 630 nm and verified by microscopic observations. Temperature of the growth medium (small dots, background) was measured using data loggers and verified by thermocouples in the actual growth media. The exponential growth curve (black line) was fit to the data by non-linear regression and the exponential growth model: a_t_ = a_0_ e^µt^, where µ is the exponential growth rate with units of h^−1^, and a_0_ and a_t_ are absorbance at time 0 and time t, respectively (Schmidt et al. 2009b). Error bars are standard deviation of measurements from four replicate cultures. Data are redrawn (with permission from the authors) from Vimercati et al. (2016).

So far, studies of eco-physiological tolerances of the genus *Naganishia* have addressed their ability to grow at low temperatures (Table 2), at moderately high salinity levels, and during extreme freeze-thaw cycles, as well as to withstand high levels of UV radiation. Table 2 summarises data from a number of sources showing the ability of *Naganishia* to grow at temperatures as low as −6°C. It is also evident from Table 2 that all *Naganishia* isolates have T_max_ values of 27°C or lower (i.e. they cannot grow at temperatures above 27°C). These T_max_ values (Table 2) indicate that all of these isolates are psychrophilic or psychrotrophic and are well adapted to function at the cold temperatures that predominate at high elevations. The only study to examine the growth of *N. friedmannii* (or any microorganism) during extreme freeze-thaw cycles was recently carried out by Vimercati et al. (2016). Vimercati et al. (2016) conducted a series of experiments with tephra soils from 6030 m.a.s.l. on Volcán Llullaillaco and showed that *N. friedmannii* was the only species to increase in relative abundance during a two-month incubation of soils at temperatures fluctuating between −10°C every night and +27°C every day. The increase in *N. friedmannii* relative abundance was even more pronounced if supplemental water was applied to the soils, but it also increased when soils were at extremely dry field levels (0.24% water). At the end of this experiment, *N. friedmannii* was isolated from these soils and then shown to be able to grow at −6°C with an optimum growth temperature of 17°C (Table 2). Even more extraordinarily, this *N. friedmannii* isolate was able to grow in liquid culture during extreme freeze-thaw cycles in the lab (Figure 4), exhibiting an overall growth rate (µ) of 0.013 h^−1^ (doubling time of 50 h), a rate equivalent to its growth rate at a constant 0°C (Vimercati et al. 2016). The experiment shown in Figure 4 was done in low volumes of growth media so that they froze every night and completely melted every day. To our knowledge, this is the first demonstration of the growth of any microbe *during* extreme freeze-thaw cycles, although activity of microbes during such cycles has long been known (reviewed in Schmidt et al. 2009a). The ability to survive and thrive within a very broad temperature range may be a characteristic widespread within the Tremellomycetes; even pathogenic *Cryptococcus gatti* and *C. neoformans* exhibit broad tolerance to a range of temperatures (Nichols et al. 2007, Garcia-Solache and Casadevall 2010), but have not been shown to be able to grow at subfreezing or fluctuating temperatures like members of the genus *Naganishia*.

**Table 2.** Growth rates, optimal temperatures (Opt), and maximum temperature (T_max_) for growth of *N. friedmannii* isolates from Llullaillaco, Socompa and Antarctic sites, and *N. vishniacii* and *N. antarcticus* from the Dry Valleys of Antarctica.

	Growth rate (h^−1^) at:					
Isolate	−6°C	−2°C	4°C	Opt	T_max_	reference
Llullaillaco	+	0.012	0.020	17°C	27°C	Vimercati et al. 2016
Saírecabur	+	+	+	nd*	25°C	Pulschen et al. 2015
Antarctica	nd	nd	+	nd	25°C	Vishniac 1985a
*N. vishniacii*	nd	nd	+	nd	25°C	Vishniac and Hempfling 1979
*N. antarcticus*	nd	nd	+	nd	15–25°C**	Vishniac and Kurtzman 1992

*not determined **Different strains of *C. antarcticus* had different T_max_ values.

*N. friedmannii* isolates have also been shown to withstand high levels of UV radiation and relatively high salt concentrations. For example, Pulschen et al. (2015) showed that an *N. friedmannii* strain could withstand levels of UV radiation on a par with the extremely radiation-resistant bacterium, *Deinococcus radiodurans*. It has also been shown recently that even non-extremophilic species in the Tremellomycetes such as the human pathogen *Cryptococcus neoformans* is among the most resistant species yet tested to gamma radiation (Khajo et al. 2011). Therefore, radiation resistance may be a wide-spread trait in the Tremellomycetes, not necessarily limited to organisms that can grow and survive in high-elevation environments.

Likewise, members of the genus *Naganishia* have been shown to be somewhat halotolerant, with growth ceasing at sodium chloride levels of 1.2–1.8 M for various strains of *N. antarticus, N. friedmannii*, and *N. vishniacii* (Vishniac and Kurtzman 1992; Pulschen et al. 2015). While these values are respectable, they do not indicate that they are halophilic and put these strains at the lower range of halotolerant yeasts. For example, Corte et al. (2006) screened 27 halotolerant yeast species that can tolerate 10% NaCl and found that growth ceased at concentrations of between 1.7 and 3.8 M, with an average of 2.5 M. In addition, none of the Antarctic *Naganishia* species can grow in the presence of 10% NaCl, whereas about 30% of the non-Antarctic species listed in Barnett et al. (2000) could grow in the presence of 10% NaCl.

Ecophysiological studies of *Naganishia* isolates indicate that some of them are metabolically versatile in terms of how many different organic compounds they can use for growth. Of particular interest is the work of Vimercati et al. (2016) who showed that the *N. friedmannii* isolate from Volcán Llullaillaco could metabolise 74% of 27 organic compounds commonly used for classifying yeast. In contrast, *N. friedmannii* and *N. consortionis* from Antarctica used 48 and 30% of the same 27 organic compounds, respectively (Barnett et al. 2000), perhaps indicating that the Antarctic isolates have a narrower metabolic niche than the Llullaillaco isolate. The metabolic versatility of the Llullaillaco isolate may indicate that this organism is an opportunitroph as defined by Polz et al. (2006), that is, an organism capable of “exploiting spatially and temporally variable resources”. Many of the substrates used for growth by this organism are plant-derived compounds (e.g. cellobiose, arbutin, salicin) indicating that it can take advantage of Aeolian transported plant material that is likely the main source of organic matter to extreme high-elevation ecosystems (Ley et al. 2004; Mladenov et al. 2012; Vimercati et al. 2016). Our working hypothesis is that *N. friedmannii* on Llullaillaco and other volcanoes are dormant during long periods of dryness and only come out of dormancy and opportunistically metabolise Aeolian organic debris following the melting of rare snow events. This sort of opportunistic strategy has also been suggested for other cold-desert microbes, especially phototrophs (Pointing 2016; Schmidt et al. 2017). More work is needed to test the opportunitroph hypothesis for *N. friedmannii* and to compare the ecological strategies of *N. friedmannii* strains from the Andes and Antarctica.

Taken together, ecophysiological studies of *N. friedmanniii* and other members of the genus *Naganishia* indicate that these are very resilient and resistant organisms, but that more work is needed to definitively prove that they can grow and function under the extreme conditions found at elevations above 6000 m.a.s.l. on volcanoes such as Llullaillaco and Socompa.

## Conclusions and future directions

Evidence presented in this review supports the hypothesis that members of the genus *Naganishia* can function as self-sustaining populations in what is probably the most extreme terrestrial environment on Earth. Their ability to withstand high levels of UV radiation and low pH values combined with their unique ability to grow during extreme freeze-thaw cycles indicates that they are adapted to live where these three stressors are continuously in play. Also, given that they are halotolerant (rather than halophilic), psychrotolerant (rather than psychrophilic), and metabolically versatile indicates that they are flexible in their response to water potential, temperature, and nutrient availability. Therefore, given the evidence available so far, our working model for why *Naganishia* species are so prevalent on these high elevation volcanoes is that they are flexible “opportunitrophs” (*cf* Polz et al. 2006; Westrich et al. 2016) that can grow during rare periods of water (from melting snow) and nutrient availability (from Aeolian inputs). However, much more work is needed to verify that *Naganishia* species are actually functioning in these high elevation soils (and at similar sites in Antarctica) and to understand how they are dispersed through the atmosphere to remote sites throughout the cryosphere.
